# HIVEP1 Is a Negative Regulator of NF-κB That Inhibits Systemic Inflammation in Sepsis

**DOI:** 10.3389/fimmu.2021.744358

**Published:** 2021-11-05

**Authors:** Hisatake Matsumoto, Brendon P. Scicluna, Kin Ki Jim, Fahimeh Falahi, Wanhai Qin, Berke Gürkan, Erik Malmström, Mariska T. Meijer, Joe M. Butler, Hina N. Khan, Tsuyoshi Takagi, Shunsuke Ishii, Marcus J. Schultz, Diederik van de Beek, Alex F. de Vos, Cornelis van ‘t Veer, Tom van der Poll

**Affiliations:** ^1^ Center for Experimental and Molecular Medicine, Amsterdam University Medical Centers, Location Academic Medical Center, University of Amsterdam, Amsterdam, Netherlands; ^2^ Department of Clinical Epidemiology, Biostatistics and Bioinformatics, Amsterdam University Medical Centers, Location Academic Medical Center, University of Amsterdam, Amsterdam, Netherlands; ^3^ Department of Medical Microbiology and Infection Prevention, Amsterdam University Medical Centers, Location Academic Medical Center, University of Amsterdam, Amsterdam, Netherlands; ^4^ Department of Neurology, Amsterdam University Medical Centers, Location Academic Medical Center, University of Amsterdam, Amsterdam, Netherlands; ^5^ Department of Disease Model, Institute for Developmental Research, Aichi Developmental Disability Center, Kasugai, Aichi, Japan; ^6^ Cluster for Pioneering Research, RIKEN, Tsukuba, Japan; ^7^ Department of Intensive Care Medicine, Laboratory of Experimental Intensive Care and Anesthesiology (L·E·I·C·A), Amsterdam University Medical Centers, Location Academic Medical Center, University of Amsterdam, Amsterdam, Netherlands; ^8^ Mahidol-Oxford Tropical Medicine Research Unit (MORU), Mahidol University, Bangkok, Thailand; ^9^ Nuffield Department of Medicine, University of Oxford, Oxford, United Kingdom; ^10^ Division of Infectious Diseases, Amsterdam University Medical Centers, Location Academic Medical Center, University of Amsterdam, Amsterdam, Netherlands

**Keywords:** HIVEP, MyD88, NF-κB, sepsis, toll-like receptors

## Abstract

Our previous work identified human immunodeficiency virus type I enhancer binding protein 1 (HIVEP1) as a putative driver of LPS-induced NF-κB signaling in humans *in vivo*. While HIVEP1 is known to interact with NF-ĸB binding DNA motifs, its function in mammalian cells is unknown. We report increased HIVEP1 mRNA expression in monocytes from patients with sepsis and monocytes stimulated by Toll-like receptor agonists and bacteria. In complementary overexpression and gene deletion experiments HIVEP1 was shown to inhibit NF-ĸB activity and induction of NF-ĸB responsive genes. RNA sequencing demonstrated profound transcriptomic changes in HIVEP1 deficient monocytic cells and transcription factor binding site analysis showed enrichment for κB site regions. HIVEP1 bound to the promoter regions of NF-ĸB responsive genes. Inhibition of cytokine production by HIVEP1 was confirmed in LPS-stimulated murine *Hivep1^-/-^* macrophages and HIVEP1 knockdown zebrafish exposed to the common sepsis pathogen *Streptococcus pneumoniae*. These results identify HIVEP1 as a negative regulator of NF-κB in monocytes/macrophages that inhibits proinflammatory reactions in response to bacterial agonists *in vitro* and *in vivo*.

## Introduction

Sepsis is caused by a deregulated host immune response to an infection and a major cause of death in hospitalized patients (1, 2). Upon infection pathogen-associated molecular pattern molecules (PAMPs) derived from the causative microorganism, such as lipopolysaccharide (LPS), interact with pattern recognition receptors on various cell types to initiate the immune response (3, 4). Toll-like receptors (TLRs) are essential components of innate immune defense during bacterial infections by virtue of their capacity to detect a large variety of PAMPs (4). TLRs (with the exception of TLR3) signal through myeloid differentiation primary response gene 88 (MyD88) to activate nuclear factor-κB (NF-κB) resulting in the production of inflammatory cytokines (3–5). Excessive NF-κB activation may cause uncontrolled inflammation, leading to tissue damage and organ failure, such as can occur in sepsis (6).

Our laboratory recently reported on changes in blood leukocyte transcriptomes after intravenous injection of LPS into healthy humans (7), a model of systemic inflammation with relevance for the early immune response to sepsis (8). Leveraging on network-based methods we identified human immunodeficiency virus type I enhancer binding protein 1 (HIVEP1), also known as Schnurri-1 (Shn-1), ZAS1, zinc finger 40, MHC enhancer binding protein 1 and positive regulatory domain II binding factor (9), as a putative driver of LPS-induced NF-kB signaling responses (7). HIVEP1 was originally isolated from a human B cell cDNA expression library that was screened for proteins binding to a DNA fragment derived from the MHC class I gene enhancer sequence 5’-TGGGGATTCCCA-3’ (10). This ĸB motif is also present in the enhancer elements of other mammalian promoters, such as those of the IL-2 receptor and *IFNB1* genes, as well as in viral promoters, including those of human immunodeficiency virus (HIV)-1 and cytomegalovirus (9). The functional consequence of HIVEP-1 binding to the ĸB motif thus far has only been shown for the interaction with the HIV-1 long terminal repeat (LTR), which results in activation of HIV-LTR gene transcription (11). To the best of our knowledge, the function of HIVEP-1 in gene transcription in immune cells has not been described. The primary objective of the current study was to investigate the functional role of HIVEP1 in the response of monocytes to bacterial agonists *in vitro* and during experimental sepsis *in vivo*.

## Materials and Methods

### Human Peripheral Blood Mononuclear Cells and Monocytes

Blood samples were obtained from 5 patients with sepsis caused by community-acquired pneumonia within 24 hours after admission to the intensive care unit of the Academic Medical Center, University of Amsterdam, the Netherlands and from 5 healthy subjects. Peripheral blood mononuclear cells (PBMCs) were isolated using Ficoll-Paque (GE Healthcare, Stockholm, Sweden) and stored in RNA protect cell reagent (Qiagen, Hilden, Germany). The Medical Ethics Committee of the Academic Medical Center approved the study (NL34294.018.10) and written informed consent was obtained from all patients (or legal representative) and healthy controls.

### Primary Monocyte Isolation and Stimulation

Buffy coats were collected from heparin anticoagulated blood by density-gradient centrifugation using Ficoll-paque plus (GE Healthcare, Stockholm, Sweden). Subsequently, monocytes were isolated using cluster-of-differentiation (CD)14^+^ magnetic beads (Miltenyi Biotec, Bergisch Gladbach, Germany, # 130-050-201) and MACS LS columns (Miltenyl Biotech). For stimulations, monocytes were placed in a 48-well plate (10^6^ per well; Greiner Bio-One, Frickenhausen, Germany) in RPMI1640 medium containing 10% fetal calf serum (FCS),100 U/ml penicillin, 2 mM L-glutamine and stimulated with medium, Pam3CSK4 (1 µg/mL; Invivogen, San Diego, CA) or LPS (from *Escherichia coli* O111:B4;100 ng/mL; Invivogen) for 4 hours. In a separate experiment, monocytes (5 × 10^5^ cells/well) were pretreated with the NF-κB inhibitor BAY11-7082 (10 µM; Tocris Bioscience, Bristol, UK) or vehicle control (dimethyl sulfoxide; Merck KGaA, Darmstadt, Germany) for 30 minutes, and then incubated in medium, with or without LPS (100 ng/mL) for 2 hours.

### Pro-Monocyte Cell Line

Pro-monocytic THP1-MD2-CD14 cells which stably express MD2, CD14 and a SEAP NF-κB reporter construct (Invivogen, San Diego, CA, # 13F03-MM) were cultured in RPMI1640 containing 10% FCS, 100 U/ml penicillin, 2 mM L-glutamine. For stimulations, cells were placed in a 24-well plate (Greiner Bio-One, Frickenhausen, Germany) at 10^6^ cells/well and incubated with medium, Pam3CSK4 (1 µg/mL) or LPS (100 ng/mL) for 4 hours. NF-kB driven SEAP was evaluated using Quanti-Blue™ (Invivogen, San Diego, CA) according to manufacturer’s protocol.

To create HIVEP1 deficient THP1-MD2-CD14 cells, plasmid pSpCas9(BB)-2A-GFP (pX458) was used (a kind gift from Dr. F. Zhang; Addgene plasmid # 48138). Target sequences for HIVEP1 were designed using the web-tool from the Zhang lab (https://zlab.bio/guide-design-resources). Oligonucleotides containing guide RNA sequences for HIVEP1 exon 3 5’-GAAGCACAAAAAGAACTTAA-3’, HIVEP-1 exon 4 5’-AACCATCTGAACTGCGTAGA-3’ and BbsI adapter sequences were ligated into BbsI digested pX458. The sequence of the constructs was verified by DNA sequencing. Both HIVEP-1 targeting plasmids were co-transfected into THP1-MD2-CD14 cells by transfection using Lipofectamine LTX and Plus reagent (Invitrogen, Waltham, MA). THP1-MD2-CD14 cells were transfected with pX458 containing control guide-1 5’-ACGGAGGCTAAGCGTCGCAA-3’ and control guide-2 5’-ATCGTTTCCGCTTAACGGCG-3 to generate controls. After 24h culture, GFP-expressing cells were sorted and plated at 1 cell per well using a SH800 Cell sorter (Sony, Tokyo, Japan). After cell expansion, chromosomal DNA was screened for gene targeting by PCR analysis and clones were further analyzed. HIVEP1 deficient and two control THP1-MD2-CD14 cell line clones were cultured in the same way as the parent THP1-MD2-CD14 cell line. Cells were seeded at 10^6^ cells/well in a 24-well plate (Greiner Bio-One, Frickenhausen, Germany) and incubated with medium, Pam3CSK4 (1 µg/mL) or LPS (100 ng/mL) for 2, 4 and 8 and lysed for RNA collection, tumor necrosis factor alpha (TNF-α), interleukin (IL)-6 (IL-6), IL-8 and IL-1β mRNA were measured by RT-qPCR analysis. To measure NF-kB driven SEAP reporter activities and TNF-α, IL-1β, IL-6, IL-8 protein levels by ELISA, THP-1 MD2-CD14 cells were seeded at 10^5^ cells/well in a 96-well plate (Greiner Bio-One, Frickenhausen, Germany) and incubated with medium, Pam3CSK4 (1 µg/mL), LPS (100 ng/mL) and heat-killed bacteria including *E.coli* (O18:K1), *Streptococcus (S.) pneumoniae* (ATCC 6303), *Klebsiella (K.) pneumoniae* (ATCC 43816) and *Pseudomonas (P.) aeruginosa* (PAO1) for 4 and 24 hours (cell to bacteria ratio, 1:10).

### Overexpression and Luciferase Assays

Human FLAG-tagged human HIVEP1 (pact empty -Flag-EP1) mammalian expression vector was used for transfection. HEK293T cells were cultured in DMEM (Lonza, Switzerland) containing 10% FCS, 100 U/ml penicillin, 2 mM L-glutamine. HEK293T cells were transfected using Lipofectamine 2000 (Invitrogen, Carlsbad, CA) according to manufacturer’s protocol after seeding and culturing the cells in culture medium without antibiotics overnight. Lipofectamine/DNA plasmid complexes were prepared in Opti-Mem (Gibco/Thermo Fisher Scientific, Waltham, MA) and incubated with the cells in Opti-Mem after removal of the culture medium. To induce HIVEP1 protein expression HEK293T cells cultured in 48-wells plates were transfected with 1 μg HIVEP1 expression vector or empty control vector complexed to 3 µl Lipofectamine in 500 μl Opti-Mem per well. After 24 hours of transfection, the cells were incubated in culture medium for 24 hours and collected for western blots.

To determine the effect of HIVEP1 on TLR mediated responses HEK293 cells stably transfected with CD14 and TLR2 (CD14/TLR2-HEK293, generously provided by Dr. D. Golenbock (University of Massachusetts Medical School, Worcester, MA, USA) were transfected with HIVEP1 expression vector or empty control vector as described above in 48-wells plates. After 24 hours of transfection, the cells were stimulated in culture medium for 2 hours with 1000 ng/ml Pam3CSK4 and lysed for RNA collection. Induction of TNF-α and A20 mRNA were measured by RT-qPCR analysis. NF-κB activity was monitored in CD14/TLR2-HEK293 cells seeded in 96 wells plates. The cells were co-transfected with 83 ng HIVEP1 or empty vector and 33 ng Firefly Luciferase NF-κB driven reporter construct and 0.7 ng Renilla Luciferase CMV driven construct to standardize for transfection efficiency. 24 hours after transfection, the cells were stimulated in culture medium for 24 hours with1000 ng/ml Pam3CSK4. The cells were lysed and NF-κB luciferase assay was performed using the DualGlo kit (Promega, Madison, Wisconsin, United States) as described (12).

### Coexpression Assay

To monitor the effect of HIVEP1 on NF-κB activity triggered by mediators downstream of TLR’s, NF-κB activation was induced in HEK293T cells by transfection with either 83 ng human tumor necrosis factor receptor associated factor (TRAF)6 or human MyD88-HA tagged (pUNO) expression vectors (Invivogen, Toulouse, France); these cells were cotransfected with the indicated amount of HIVEP1 or control plasmid and 33 ng of NF-kB-Luciferase and 0.7 ng of Renilla-Luciferase constructs in a final volume of 100 µl Opti-Mem. The total amount of DNA expression vectors was kept constant using empty vector control plasmid. After 24 hours of co-transfection, the cells were lysed and NF-κB luciferase assay was performed as described above; cells transfected with MyD88 or TRAF6 were not further stimulated with TLR agonists.

To determine the effect of HIVEP-1 co-transfection on MyD88 and TRAF6 expression, HEK293T cells cultured in 6 wells plates were cotransfected with 2 μg HIVEP1 or control plasmid and either 2 μg human TRAF6 or human MyD88-HA tagged (pUNO) expression vector (Invivogen, Toulouse, France) complexed to 10 ul Lipofectamine. After 24 hours of transfection, the cells were incubated in culture medium for 24 hours and collected for western blots.

### Assays

Real-time quantitative RT-PCR (qPCR) was performed to evaluate mRNA expressions. Total RNAs were extracted with RNA isolation kits (Nucleospin, MACHEREY-NAGEL, Germany) according to manufacturer’s protocol and reverse transcribed with oligo(dT) primer and moloney murine leukemia virus reverse transcriptase (Promega, Madison, WI). RT-qPCR analysis was performed using the SensiFast Sybr green mix (Bioline, London, UK) on a LightCycler system (LC480, Roche Applied Science, Penzberg, Germany). LinRegPCR software was used to analyze the data. Primers used are depicted in **Supplementary Table 1**. Human TNF-α, IL-6 and IL-8, and murine TNF-α, IL-6 and CXCL1 protein levels were measured by ELISA (all Duo set R&D systems, Minneapolis, MN).

### Western Blot Analysis

Western blot analysis was performed as described previously with modification (12). Briefly, cells were lysed using cell lysis buffer (Cell Signaling Technology, Danvers, MA) containing a 5-times higher concentration of the Halt protease and phosphatase inhibitor cocktail (Thermo Fisher Scientific, Waltham, MA) than recommended by the supplier to prevent HIVEP1 degradation. The samples were boiled at 95°C for 5 min in Laemmli sample buffer containing 5% β-mercaptoethanol. For the detection of the large HIVEP1 protein (approximately 296kDa, 269kDa and 259kDa respectively) 6% SDS-polyacrylamide gels were used, and separated proteins were transferred to PVDF membranes. Blots were incubated with rabbit polyclonal anti-FLAG antibody (Sigma-Aldrich, St. Louis, MO, # F7425;1:1000) or anti-MyD88 antibody (Cell Signaling, Danvers, Massachusetts; # 3699 1:1000) or anti-TRAF6 antibody (Cell Signaling, Danvers, Massachusetts; # 8028 1:1000) and horseradish peroxidase–conjugated secondary anti-rabbit-IgG antibody (Cell Signaling, Danvers, Massachusetts; # 7074S 1:1000) to detect FLAG-tagged HIVEP1 protein or MyD88 or TRAF6. The membranes were imaged using LumiLight Plus ECL (Roche, Basel, Switzerland) on a LAS 4000 chemiluminescence imager (GE Healthcare Bio-Sciences, Piscataway, NJ).

### RNA Isolation and Sequencing

Total RNA was isolated from HIVEP1 deficient THP1-MD2-CD14 cells (n=4) and control cells (n=4), treated with or without 100ng/mL LPS for 2 and 8 hours, using the RNeasy mini kit according to the manufacturer’s instructions (Qiagen, Hilden, Germany). RNA quality was assessed by bioanalysis (Agilent), with all samples having RNA integrity numbers > 7. Total RNA concentrations were determined by Qubit^®^ 2.0 Fluorometer (Life Technologies, Carlsbad, CA, USA). RNA-sequencing libraries were prepared from 200ng total RNA using KAPA RNA Hyperprep with RiboErase (Roche) library kits and sequenced using the Illumina HiSeq4000 instrument (Illumina) to generate single reads (50bp). Quality was assessed by means of the FastQC method (v0.11.5; http://www.bioinformatics.babraham.ac.uk/projects/fastqc/). Trimmomatic version 0.36 (13) was used to trim Illumina adapters and poor-quality bases (trimmomatic parameters: leading=3, trailing=3, sliding window=4:15, minimum length=40). The remaining high-quality reads were used to align against the Genome Reference Consortium human genome build 38 (GRCh38) (14). Mapping was performed by HISAT2 version 2.1.0 (15) with parameters as default. Count data were generated by means of the HTSeq method (16), and analyzed using the DESeq2 method (16) in the R statistical computing environment (R Core Team 2014. R: A language and environment for statistical computing. R Foundation for Statistical Computing, Vienna, Austria). Statistically significant differences were defined by Benjamini & Hochberg adjusted probabilities < 0.05. Canonical signaling pathways and biofunctions were inferred using Ingenuity Pathway Analysis (QIAGEN bioinformatics) specifying human species and ingenuity database as reference. All other parameters were default. Benjamini-Hochberg adjusted Fisher’s test p < 0.05 demarcated significance. Transcription factor binding site enrichment analysis was done using the Opossum database and methods (17), specifying the first 2000 bases upstream of the transcription start sites. All other parameters were default.

### Public Gene Expression Data

For determining expression of *HIVEP1* mRNA in monocytes from patients with sepsis we used publicly available data deposited on Gene Expression Omnibus (GEO) under the accession number GSE46955 (18). In brief, monocytes were purified from PBMCs by using CD14+ monocyte isolation kit from Miltenyi Biotec (Bergisch Gladbach, Germany; >90% pure) from 8 patients with sepsis with microbiologically confirmed Gram-negative bacteremia secondary to urinary tract infection within 4 hours after admission and 5 age-matched healthy controls. RNA expression was analyzed by microarray (Illumina Human Ref8 v2 Beadchips). For this analysis we used normalized gene expression values provided in GSE46955.

### Chromatin Immunoprecipitation Assay

Chromatin immunoprecipitation (ChIP) analysis was performed using ChIP-IT Express kit (Active Motif, Carlsbad, CA) following manufacturer’s instructions. For CHIP-RT-qPCR analysis, 1.5 x10^7^ HEK293T cells were seeded into 15-cm culture dishes with DMEM containing 10% FCS and 2 mM L-glutamine without antibiotics overnight prior to the transfection. 30 µg of HIVEP1 expression vector or empty vector was prepared for each dish. Transfection was conducted in the same manner as described above. Briefly, 15mL of Opti-Mem with DNA/lipofectamine complex was added into each dish. 24 hours after transfection, the cells were collected for CHIP. The cells were treated with 1% paraformaldehyde for 10 min at room temperature to crosslink DNA/protein adducts. Extracted chromatin was sheared by sonication using a Diagenode BioRuptor (10 pulses of 20 seconds each, with a 30 second rest between each pulse) (Diagenode Diagnostics, Seraing (Ougrée), Belgium) into fragments from 200-800 bp length and immunoprecipitated with magnetic beads using 3 μg mouse monoclonal anti-FLAG^®^ M2 antibody (Sigma-Aldrich, St. Louis, MO, # F1804-1MG) overnight. The magnetic beads were washed with CHIP buffer in the kit, the samples were reverse cross-linked and proteins were digested with proteinase K. RT-qPCR amplification was performed using 3 μl of total DNA and immunoprecipitated DNA using *TNF* and *TNFAIP3* promoter primers spanning the NF-κB binding regions (**Supplementary Table 1**).

#### Mice

*Hivep1^-/-^* mouse embryos (C57BL6/JJmsSlc background) were purchased from RIKEN BioResource center (BRC) (Ibaraki, Japan; RBRC0 1172). Deletion of part of exon 4 and insertion of NEO were confirmed by southern blot and PCR analysis with genomic DNA (**Supplementary Figures 1A, B**). Genotyping PCR was performed using primers in *hivep1* exon3_4 with genomic DNA to verify deletion of *hivep1* (**Supplementary Figure 1C**). Mice were bred under specific pathogen-free conditions in the animal facility of the Academic Medical Center in Amsterdam. Experiments were carried out in accordance with the Dutch Experiment on Animals Act and with permission from the Animal Care and Use Committee of the University of Amsterdam (Permission number: DIX288BY and DIX288CC).

### HIVEP1 Deficient Mouse Macrophages

Bone marrow-derived macrophages (BMDMs) were cultured as described previously with some modifications (19). In short, femurs and tibia were collected from 8-12 week old *hivep1^-/-^* mice and wild-type littermates. BMDMs were collected by flushing bone shafts with ice-cold sterile PBS on day 0. BMDMs were cultured in RPMI1640 medium containing 10% FCS, 100 U/ml penicillin, 2 mM L-glutamine with15% L-929 cell-conditioned medium (LCM). On day 9, BMDMs in medium with15% LCM were seeded at 10^6^ cells/well in a 24 well plate (Greiner Bio-One, Frickenhausen, Germany) for RNA harvest or at 10^5^ cells/well in a 96-well plate (Greiner Bio-One, Frickenhausen, Germany) for cytokine release, allowed to attach overnight and then incubated with medium or LPS (100 ng/mL) for 2, 8 or 24 hours.

### Zebrafish Embryo Experiments

Zebrafish handling, embryo care and microinjections were performed as described previously with some modifications (20). Embryos from the transparent adult *casper* mutant zebrafish (21) line were used. To transiently block the expression of HIVEP1 we used an antisense morpholino (MO) knockdown approach. A HIVEP1 splice-modifying MO (H1MO) targeting exon-intron junction 6 to block HIVEP1 pre-mRNA splicing, thereby altering protein synthesis, was designed by Gene Tools (Gene tools LLC, Philomath, OR) [H1MO against HIVEP1 exon-intron junction 6 sequence: 5’-CAGTGTGTTTGAGACTGAACCCACC-3’]. To determine the optimal concentration of H1MO morpholino at which injected embryos survive and without developmental delay or morphological malformations, such as somite deformities, we performed a dose-response experiment. A standard mismatch control morpholino (SCMO) was used as a negative control [SCMO: 5’-CCTCTTACCTCAGTTACAATTTATA-3’]. The optimal concentration of the H1MO at which the embryos survive without abnormalities was 0.5 mM and the injection volume 1 nL. The MO solutions were injected into the yolk of one- to four-cell stage embryos. RT-qPCR was performed to analyze the mRNA product in H1MO knockdown and SCMO injected embryos and the RT-qPCR product(s) were analyzed by gel electrophoresis. Splice modification is considered successful if this results in mobility shift and partial loss of the wild-type transcript (**Supplementary Figures 2A, B**). H1MO and SCMO treated zebrafish embryos were injected at 2 days post fertilization (dpf) with ~200 colony forming units (CFU) of *Streptococcus pneumoniae* D39 in the caudal vein, resulting in a systemic infection. The experimental protocol is shown in **Supplementary Figure 2C**. Mortality rates were determined in three independent experiments (n = 20 embryos per group in each experiment) by monitoring live and dead embryos at 24, 36 and 48 hours post infection. For RNA analysis zebrafish embryos were collected at 2 or 24 hours post infection and anaesthetized with 0.02% Tricaine (n=10 per group). For every 10 embryos 350 µl of lysis buffer (Nucleospin, MACHEREY-NAGEL, Duren, Germany) was added according to manufacturer’s protocol and repeatedly drawn through a 26-gauge needle into a syringe and expelled, until all tissue was homogenized. TNF-α, IL-1β, IL-8a, IL-8b mRNA expressions were measured by qPCR (for primers see **Supplementary Table 1**). Zebrafish were handled in accordance with the local animal welfare regulations and standard protocols. All experimental procedures were followed by international guidelines specified by the EU Animal Protection Directive 86/609/EEC.

### Statistical Analysis

Data are presented as means with standard error of the mean. Differences between groups were analysed by Mann-Whitney U test. Survival curves were compared using log-rank statistics and a p-value < 0.05 defined significance. Analyses were performed with JMP Pro 13.0 for Windows (SAS Institute Inc., Cary, NC) or GraphPad Prism 7.0 (GraphPad Software Inc, San Diego, CA).

## Results

### HIVEP1 mRNA Expression Is Increased in Monocytes of Sepsis Patients and Upon Stimulation With Bacterial Agonists *In Vitro*

**Figure 1:**
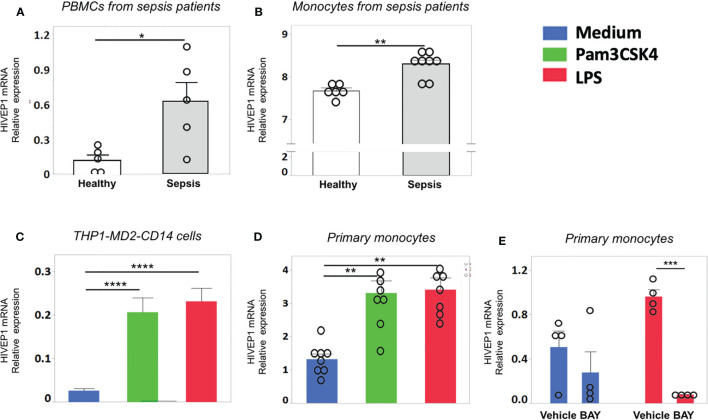
HIVEP1 mRNA expression is increased in patients with sepsis and upon stimulation with bacterial agonists *in vitro*. HIVEP1 mRNA in PBMCs **(A)** and monocytes **(B)** from patients with sepsis (5 and 8 individual donors, respectively) upon admission to the intensive care unit and in healthy subjects (5 and 6 individual donors, respectively). The monocytes data was obtained from GEO database (GSE46955). **(C)** HIVEP1 mRNA in human monocytic wild type THP1-MD2-CD14 cells incubated with medium, PAM3CSK4 (1 µg/ml) or LPS (100 ng/mL) for 4 hours. **(D)** HIVEP1 mRNA in primary human monocytes incubated with medium (control), PAM3CSK4 (1 µg/ml) or LPS (100 ng/mL) for 4 hours. For **(C)**, three experiments with four replicates were conducted. For **(D)**, two experiments each with 4 independent donors (8 individual donors in total) were conducted. Pooled data from three **(C)** or two **(D)** independent experiments are displayed. **(E)** HIVEP1 mRNA in primary human monocytes (from 4 individual donors) incubated with medium or LPS (100 ng/mL) for 2 hours in the presence or absence of the NFκB inhibitor BAY11-7082 (or vehicle) added 30 minutes prior to LPS. HIVEP1 mRNA expression was normalized to HPRT mRNA **(A, C, D, E)**. For **(B)** normalized gene expression values were used as provided in GSE46955. Data shows means ± SEM with individual data shown as dots. *P < 0.05, **P < 0.01, ***P < 0.001, ****P < 0.0001.

Patients with sepsis due to community-acquired pneumonia showed increased HIVEP1 mRNA expression in peripheral blood mononuclear cells purified from blood obtained within 24 hours after admission to the Intensive Care Unit, when compared with those from healthy controls (**Figure 1A**). Likewise, patients with blood culture positive (Gram-negative bacteremia) urosepsis had increased monocyte HIVEP1 mRNA expression when compared to age-matched controls (**Figure 1B**). Human monocytic THP1 cells with stable overexpression of MD2, CD14 and a NF-κB secreted embryonic alkaline phosphatase (SEAP) reporter construct (THP1-MD2-CD14 cells) and primary human monocytes harvested from healthy donors responded with an upregulation of HIVEP1 mRNA upon stimulation with PAM3CSK4 (TLR2 ligand) or LPS (TLR4 ligand) (**Figures 1C, D**). The induction of HIVEP1 mRNA in LPS stimulated monocytes was prevented by the NF-κB inhibitor BAY11-7082 (**Figure 1E**). Together, these data indicate that human monocytes increase HIVEP1 mRNA upon exposure to bacterial agonists in a NF-κB dependent manner and that THP1-MD2-CD14 cells are a suitable model to study the functional role of HIVEP1.

### HIVEP1 Overexpression Inhibits NF-κB Activity Upon TLR Stimulation

**Figure 2:**
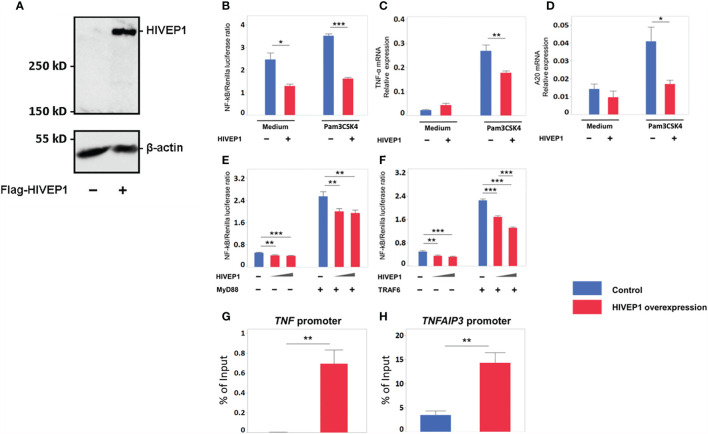
Overexpression of HIVEP1 inhibits NF-κB activity upon Toll-like receptor stimulation and binds to the TNF and TNFAIP3 promoter regions. **(A)** HIVEP1 protein expression in HEK293T cells transfected with HIVEP1 expression vector (1 µg) or control plasmid **(B–D)** CD14/TLR2 HEK293T cells were transfected with 83 ng of HIVEP1 or control vector together with 33 ng of NF-kB and 0.7 ng of Renilla constructs and incubated with medium or PAM3CSK4 (1 µg/mL); overexpression of HIVEP1 was associated with reduced activity of NF-κB **(B)**, and reduced expression of TNF-α mRNA **(C)** and A20 mRNA **(D)**. HEK293T cells transfected with MyD88 **(E)** or TRAF6 **(F)** (or control cells) were co-transfected with increasing doses of HIVEP1 expression vector (0, 30 or 150ng); these MyD88 or TRAF6 transfected cells were not stimulated with PAM3CSK4; overexpression of HIVEP1 was associated with a dose-dependent inhibition of MyD88 or TRAF6 driven NF-κB activity. mRNA expression was normalized to HPRT mRNA. Data shows means ± SEM. **(G, H)** HEK293T cells were transfected with 30 µg of HIVEP1 expression vector or control plasmid. 24 hours after transfection, the cells were collected for CHIP analysis. RT-qPCR amplification was performed with total DNA and immunoprecipitated DNA using *TNF* and *TNFAIP3* promoter primers spanning the NF-κB binding regions. Data shows means ± SEM and presented as a percentage of input DNA. Data shown were pooled from two **(A,B,C)**, three **(E, F)** independent data sets (four replicates samples for each condition within each experiment) and three **(G, H)** independent data sets (two duplicate samples for each experiment)*P < 0.05, **P < 0.01, ***P < 0.001.

To obtain a first insight into the functional role of HIVEP1, we transfected HEK293T cells with a plasmid for FLAG-tagged human HIVEP1 expression; control cells were transfected with an empty expression vector. HIVEP1 transfected cells showed specific expression of HIVEP1, as determined by western blot (**Figure 2A**). To define the effect of HIVEP1 overexpression on NF-κB activity upon TLR stimulation, we co-transfected TLR2 expressing HEK293T cells containing a NF-κB reporter construct with HIVEP1 and stimulated these cells with PAM3CSK4. Overexpression of HIVEP1 inhibited NF-κB activity induced by TLR2 stimulation (**Figure 2B**). To confirm the effect of HIVEP1 on NF-κB activity, we measured the expression of two NF-κB responsive genes, *TNF* (encoding tumor necrosis factor (TNF)-α] and *TNFAIP3* [encoding A20). Indeed, overexpression of HIVEP1 reduced induction of TNF-α and A20 mRNA upon stimulation of TLR2 (**Figures 2C, D**). To determine at which level of the TLR signaling cascade HIVEP1 influenced NF-κB activity, we co-transfected HEK293T cells with either MyD88 or TNF receptor associated factor (TRAF) 6 and HIVEP1 and determined NF-κB activity. Overexpression of HIVEP1 inhibited NF-κB activity driven by both MyD88 and TRAF6 (**Figures 2E, F**). We confirmed that co-transfection of HIVEP-1 does not affect MyD88 or TRAF6 expression (**Supplementary Figures 4A, B**). This suggests that HIVEP1 may act downstream of TRAF6. To clarify the mechanism of inhibition of NF-κB activity by HIVEP1, chromatin immunoprecipitation (ChIP)-qPCR analysis was performed using primers spanning the NF-ĸB binding sites within the *TNF* and *TNFAIP3* promoter regions. This analysis showed that HIVEP1 binds to *TNF* and *TNFAIP3* promoters in HIVEP1 transfected HEK293T cells (**Figures 2G, H**), suggesting that HIVEP1 inhibits NF-κB activity by competition for NF-κB binding sites in *TNF* and *TNFAIP3* promoter regions.

### *HIVEP1* Deletion Increases NF-kB Activity Upon TLR Stimulation

To obtain insight into the functional role of endogenous HIVEP1, we generated a *HIVEP1* deficient THP1-MD2-CD14 cell line targeted at exon 3 and 4 of *HIVEP1* by CRISPR/Cas9 and THP1-MD2-CD14 control clone without *HIVEP1* deletion (**Supplementary Figures 3A, B**).

**Figure 3:**
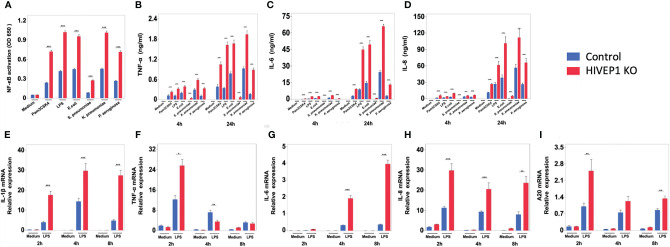
HIVEP1 deficient human monocytic cells show increased NFκB activity and proinflammatory cytokine production upon stimulation with TLR ligands or bacteria. HIVEP1 deficient and control human monocytic THP1-MD2-CD14 cells were stimulated with PAM3CSK4 (1 µg/mL), LPS (100 ng/mL), heat-killed *E*. *coli*, *S. pneumoniae*, *K*. *pneumoniae* or *P. aeruginosa.* HIVEP1 deficiency was associated with **(A)** enhanced NFκB activity (24-hour stimulation), **(B–D)** enhanced TNF-α, IL-6 and IL-8 release (4 and 24 hour stimulation) and **(E–I)** increased IL-1β, TNF-α, IL-6, IL-8 and A20 mRNA expression (normalized to HPRT mRNA). Data shows means ± SEM and were pooled from two **(E–I)** and three **(A–D)** independent data set (n = 4 for each condition within each experiment). *P < 0.05, **P < 0.01, ***P < 0.001.

**Figure 4:**
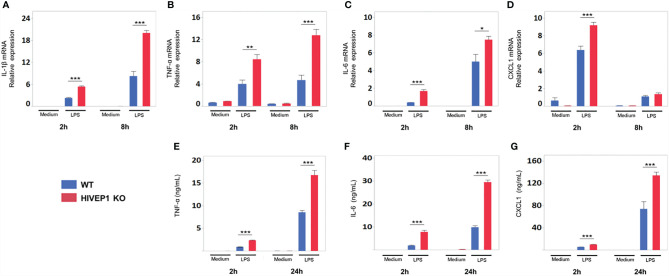
HIVEP1-deficient murine macrophages show increased proinflammatory cytokine production upon stimulation with LPS. Bone marrow derived macrophages from *hivep1^-/-^* mice and wild type littermates were stimulated with LPS (100 ng/mL). *Hivep1^-/-^* macrophages showed increased expression of TNF-α, IL-1β, IL-6, CXCL1 mRNA upon LPS stimulation **(A–D)**, as well as enhanced TNF-α, IL-6 and CXCL1 protein release **(E–G)**. Data shows means ± SEM and were pooled from three independent data set [n = 4 **(A–D)** or n = 12 **(E–G)** for each condition within each experiment)]. *P < 0.05, **P < 0.01, ***P < 0.001.

LPS stimulation upregulated HIVEP1 mRNA expression in control cells, but not in *HIVEP1* deficient cells, confirming the successful ablation of *HIVEP1* after CRISPR/Cas9 (**Supplementary Figure 3C**). When compared to control cells, *HIVEP1* deficient cells showed enhanced NF-κB activity (**Figure 3A**) and increased release of TNF-α, IL-6 and IL-8 upon stimulation with PAM3CSK4 (TLR2), LPS (TLR4), *Escherichia coli*, *Streptococcus pneumoniae*, *Klebsiella pneumoniae* or *Pseudomonas aeruginosa* for 4 hours or 24 hours (**Figures 3B–D**). In accordance, HIVEP1 deficient cells showed increased mRNA expression of IL-1β, TNF-α, IL-6, IL-8 and A20 upon stimulation with LPS (**Figures 3E–I**). In order to confirm that HIVEP1 negatively regulates NF-κB responsive gene expression, we stimulated bone marrow derived macrophages harvested from *hivep1^-/-^* mice and wild-type littermate control mice with LPS. LPS stimulation induced HIVEP1 gene expression in wild-type but not in *hivep1^-/-^* macrophages (**Supplementary Figure 1D**). *Hivep1^-/-^* macrophages showed increased expression of IL-1β, TNF-α, IL-6 and CXCL1 mRNA upon LPS stimulation (**Figures 4A–D** **)**, as well as enhanced TNF-α, IL-6 and CXCL1 protein release (**Figures 4E–G**).

### Quiescent and LPS-Stimulated *HIVEP1* Deficient Pro-Monocytic Cells Exhibit Profound Transcriptomic Changes

**Figure 5:**
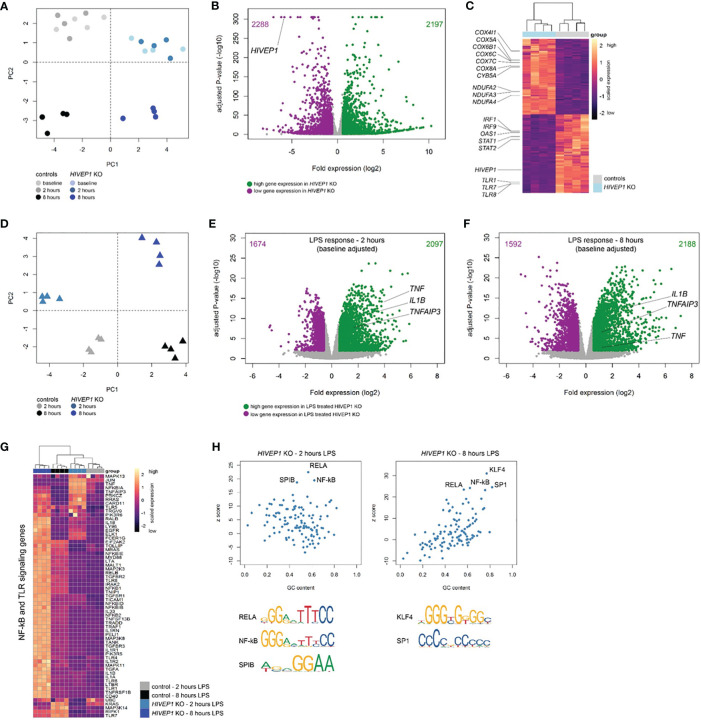
RNA-sequencing of HIVEP1 deficient THP-1 cells before and after LPS stimulation. **(A)** Principal component plot of 30,013 genes per sample showing clustering of HIVEP1 deficient cells and controls at baseline and after 2 and 8 hours culturing in medium. **(B)** Volcano plot depicting genome-wide changes in gene expression of HIVEP1 deficient cells relative to controls at baseline. Green dots denote high expression genes (Benjamini-Hochberg (BH) adjusted p < 0.01 and fold change ≥ 1.5); purple dots depict low expression genes (BH adjusted p < 0.01 and fold change ≤ -1.5). **(C)** Unsupervised heatmap plot of 4485 significantly altered genes at baseline illustrating genes involved in oxidative phosphorylation, interferon signaling and pattern recognition receptors. **(D)** Principal component plot (30,013 genes) of LPS-treated (2 and 8 hours) HIVEP1 deficient cells and controls showing clear clustering. **(E)** Volcano plot depicting genome-wide changes in LPS-induced gene expression (corrected for baseline differences) of HIVEP1 deficient cells relative to controls at 2 hours. **(F)** Volcano plot illustrating transcriptomic differences in LPS-induced gene expression (baseline corrected) of HIVEP1 deficient cells relative to controls at 8 hours. **(G)** Heatmap representation of genes involved in Toll-like receptor and NF-kB signaling in LPS-treated samples. **(H)** Dot plots depicting z-scores against GC content of transcription factor binding site enrichment analysis.

To further understand HIVEP1 function we incubated HIVEP1 deficient and control THP1-MD2-CD14 cells with LPS (100 ng/ml) or medium for 2 or 8 hours and analysed their genome-wide transcriptomes by bulk RNA-sequencing. Considering quiescent cells, HIVEP1 deficiency resulted in substantial changes in gene expression with clear clustering of cells by genotype (**Figure 5A**). Comparing unstimulated HIVEP1 deficient cells to control cells and considering multiple comparison adjusted *p*-values < 0.01 with fold change ≥ 1.5 or ≤ -1.5 identified 4485 significantly altered genes (2197 elevated; 2288 reduced) (**Figures 5B, C**). As expected, *HIVEP1* expression levels were robustly reduced in deficient cells. Pathway analysis of significantly altered genes revealed associations with various canonical signaling pathways, including high expression of metabolic genes (e.g. oxidative phosphorylation) and low expression of various inflammatory, cell-to-cell crosstalk and cell development pathways (e.g. interferon signaling, PRR signaling, communication between innate and adaptive immune cells, IL-10 signaling) (**Figure 5C** and **Supplementary Table 2**). This indicates that quiescent *HIVEP1* deficient cells have altered transcriptomes suggesting profound changes to their functional state. Next, we sought to evaluate the LPS response in *HIVEP1* deficient cells relative to controls. Principal component analysis of LPS-induced transcriptomes obtained at 2 and 8 hours post-stimulation showed clear clustering of *HIVEP1* deficient cells and controls (**Figure 5D**). By fitting a linear model that adjusted for the changes in gene expression that were observed in quiescent cells (baseline), we found 3771 (2097 high expression; 1674 low expression) and 3780 (2188 high expression; 1592 low expression) significantly altered genes in *HIVEP1* deficient cells treated with LPS for 2 and 8 hours, respectively (**Figures 5E, F**). Notably, LPS-induced *TNF*, *IL1B* and *TNFAIP3* (*A20*) expression was significantly higher in *HIVEP1* deficient cells relative to controls at both 2 and 8 hours post-stimulation. Pathway analysis of significantly altered genes at both time points identified associations with various innate immune, cell morphology, mobility and development pathways (**Supplementary Tables 3, 4**). In particular, genes involved in TLR and NF-kB signaling were over-expressed in *HIVEP1* deficient cells, for example *TLR4*, *TLR5*, *TOLLIP*, *NFKB1*, *NFKB2*, *IL1RN* and *IL1A* (**Figure 5G**). Moreover, transcription factor binding site analysis (single-site) showed high enrichment for κB site regions (NF-kB motif) as well as RELA, SPIB, SP1 and KLF4 DNA-binding motifs in the HIVEP1 altered genes (**Figure 5H**).

### HIVEP1 Knockdown Increases Systemic Inflammation and Mortality During Sepsis in Zebrafish Embryos

**Figure 6:**
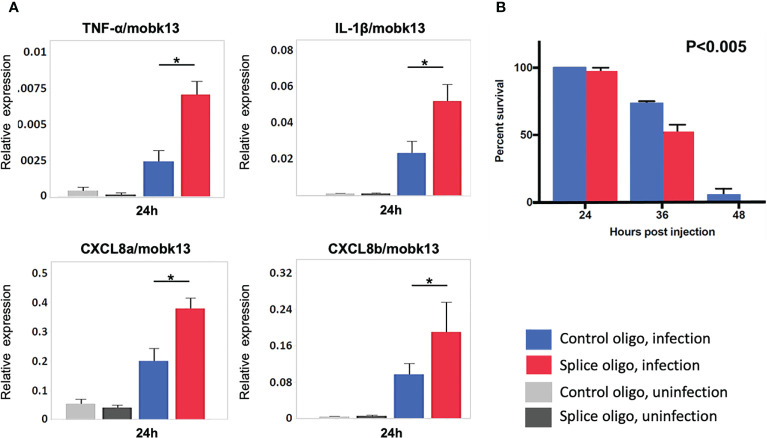
HIVEP1 knockdown increases systemic inflammation and impairs survival in zebrafish embryos with sepsis. **(A)** RNA expression of inflammatory cytokines normalized to mobk13 measured by RT-qPCR. Zebrafish embryos (n = 10) were pooled into one biological replicate and five independent experiments were performed. Data shows mean ± SEM **(B)** The survival rate was significantly lower in the HIVEP1 splice morpholino injected group than that in control (n = 20 per group). Data shows means ± SEM. Experiment performed in triplicate. Survival rates were compared using log-rank statistics. *P < 0.005.

To study the role of HIVEP1 during bacterial sepsis *in vivo*, we treated zebrafish embryos with antisense morpholinos directed against HIVEP1 or a mismatch control morpholino prior to systemic infection with *Streptococcus (S.) pneumoniae*. This pathogen is a common causative agent in human sepsis (1, 22) and zebrafish represent a suitable model system to study the pathophysiology of infections caused by *S. pneumoniae* (20). The HIVEP1 specific morpholino H1MO was designed to target exon-intron junction to induce exon6 skipping. PCR analysis of HIVEP1 mRNA products showed that only a small percentage of the normal splice product was present, together with alternatively spliced HIVEP1 mRNA products after H1MO injection, suggesting that H1MO successfully altered HIVEP1 expression (**Supplementary Figures 2A**–**C**) showing the experimental design). Injection of specific or control morpholinos did not modify cytokine/chemokine mRNA expression in uninfected zebrafish (**Figure 6A**). In zebrafish with sepsis due to inoculation with *S. pneumoniae* with the morpholino directed against HIVEP1 was associated with increased mRNA expression of TNF-α, IL-1β, CXCL8a and CXCL8b, as well as enhanced mortality (**Figures 6A, B**).

## Discussion

HIVEP1 is a 300-kDa cellular protein with two widely spaced zinc finger domains. While it has been known for over 30 years that each of these domains can bind to NF-ĸB interactive DNA motifs (23, 24), the functional consequences of this interaction have thus far not been described in immune cells. We here report enhanced HIVEP1 mRNA expression in monocytes from patients with sepsis, as well as monocytes stimulated by a variety of TLR agonists and bacteria. By using both overexpression and gene deletion approaches we show that HIVEP1 inhibits NF-ĸB activity and induction of NF-ĸB responsive genes. RNA sequencing demonstrated large changes in the transcriptome of HIVEP1 deficient monocytic cells and transcription factor binding site analysis showed strong enrichment for κB site regions. ChIP-qPCR analysis confirmed HIVEP1 binding to the promoter regions of NF-ĸB responsive genes. Inhibition of cytokine production by HIVEP1 was confirmed in LPS-stimulated murine *Hivep1^-/-^* macrophages *in vitro* and in HIVEP1 knockdown zebrafish exposed to the common sepsis pathogen *S. pneumoniae in vivo*. Together, these results identify HIVEP1 as a negative regulator of NF-κB in monocytes/macrophages that inhibits proinflammatory reactions in response to bacterial agonists *in vitro* and *in vivo*.

The current study was instigated by an earlier investigation from our laboratory that identified *HIVEP1* as a hub gene in the NF-κB signaling module induced in blood leukocytes after intravenous injection of LPS into healthy humans (7). Genome-wide analysis of the blood leukocyte transcriptome revealed the NF-κB signaling module as one of the top upregulated pathways upon LPS administration. A network-based bioinformatics analysis of transcriptional relationships predicted HIVEP1 as a LPS-induced modulator of the NF-kB signaling module triggered by intravenous LPS (7). The current study provides proof of this earlier assumption. Our finding of upregulation of HIVEP1 mRNA in mononuclear cells upon specific stimulation of TLR2 or TLR4, and after exposure to a variety of bacteria, is consistent with an earlier study reporting increased HIVEP1 mRNA levels in mouse bone marrow derived macrophages infected with *Mycobacterium tuberculosis* (25). We show that induction of *HIVEP1* in LPS-stimulated monocytes is regulated by NF-κB, suggesting that HIVEP1 represents a negative feedback mechanism upon activation of NF-κB. Stimulation of TLR2 or TLR4 results in NF-κB activation *via* a MyD88-TRAF6 dependent route (3, 26). TRAF6 initiates NF-kB activation *via* canonical phosphorylation of IKK complex by TAK1, leading to the production of inflammatory cytokines (26). By using a gain-of-function approach we showed that HIVEP1 may act downstream of TRAF6 to inhibit NF-κB. Indeed, CHIP-qPCR showed binding of HIVEP1 to the promoter regions of *TNF* and *TNFAIP3* which is in agreement with earlier studies reporting HIVEP1 binding to the ĸB motif in the enhancer elements of the IL-2 receptor and *IFNB1* genes (9). Importantly, we here show that this interaction results in inhibition of proinflammatory cytokine production and confirmed this by using a loss-of-function approach across three species, i.e., human and mouse mononuclear cells and intact zebrafish. RNA-sequencing analyses of LPS-stimulated human *HIVEP1* deficient monocytic cells confirmed the inhibitory role of HIVEP1 in expression of genes involved in TLR and NF-κB signaling, and transcription factor binding site analysis revealed strong enrichment for κB site regions. *HIVEP1* knockdown impacted a variety of additional pathways in human monocytic cells, which could guide future research on the functional role of HIVEP1. Consistent with the current data, promoter-level expression profiling suggested a role for HIVEP1 in macrophage activation (27). Interestingly, genetic variation at the *HIVEP1* locus was associated with an increased risk for venous thrombosis (28) and enhanced inflammation has been implicated in the pathogenesis of thrombosis (29).

Our study has several limitations. Overexpression of HIVEP1 was done in HEK293T cells; likewise, experiments to determine whether HIVEP1 acts up- or downstream of MyD88 and TRAF6, and to investigate the interaction with HIVEP1 with the *TNF* and *TNFAIP3* promoters, were performed in this cell line. It would be valuable to confirm these results in monocytes. Overexpression of HIVEP1 may result in artificial effects, especially considering that endogenous HIVEP1 protein levels are low. Most likely as a consequence of the latter, we were unable to detect endogenous HIVEP1 protein levels in THP1 cells and monocytes using a variety of antibodies. NFĸB activity was determined using a gene reporter system and measurement of NFĸB responsive genes; additional readouts of NFĸB activity would have strengthened the conclusion that HIVEP1 is a negative regulator of NFĸB.

In conclusion, we here demonstrate that HIVEP1 acts as repressor of NF-kB activity induced by bacterial agonists and that HIVEP1 deficiency exacerbates inflammation in sepsis. These results suggest HIVEP1 as a key regulator of NF-κB activity in the pathogenesis of sepsis.

## Data Availability Statement

Sequence libraries in this study are publicly available through the National Center for Biotechnology Information (NCBI) gene expression omnibus (GEO) (http://www.ncbi.nlm.nih.gov/geo/) with accession identifier GSE162757.

## Ethics Statement

The studies involving human participants were reviewed and approved by the Dutch Central Commission for Human bound Research (CCMO) (study identifier NL34294.018.10). The patients/participants provided their written informed consent to participate in this study. The animal study was reviewed and approved by the Institutional Animal Care and Use Committee of the University of Amsterdam (DIX288BY and DIX288CC).

## Author Contributions

HM performed the experiments, analyzed the data, and wrote the first draft of the manuscript. KJ and DB contributed to the experiments with zebrafish. FF, WQ, EM, and MTM performed part of the experiments. BG contributed to the interpretation of the data. TT contributed to the interpretation of the data and knockout mice. SI contributed to the knockout mice. JB. and HK performed part of the analyses. BS analyzed the RNA-seq data, contributed to the interpretation of the data and revised the manuscript for important intellectual content. AV and CV supplied reagents and contributed to the interpretation of the data and revised the manuscript for important intellectual content. TP supervised the project and completed the manuscript. All authors read and approved the final version of the manuscript.

## Funding

HM is funded by SENSHIN Medical Research Foundation. EM is funded by Wenner-Gren Foundations (FT2020-0003) and Stiftelsen P E Lindahls stipendiefond Medicine (LM20170016).

## Conflict of Interest

The authors declare that the research was conducted in the absence of any commercial or financial relationships that could be construed as a potential conflict of interest.

## Publisher’s Note

All claims expressed in this article are solely those of the authors and do not necessarily represent those of their affiliated organizations, or those of the publisher, the editors and the reviewers. Any product that may be evaluated in this article, or claim that may be made by its manufacturer, is not guaranteed or endorsed by the publisher.
