# Supplementary material for: HIVEP1 Is a Negative Regulator of NF-κB That Inhibits Systemic Inflammation in Sepsis
Source: Front Immunol. 2021 Nov 5;12:744358. doi: 10.3389/fimmu.2021.744358 (PMC8602905; doi:10.3389/fimmu.2021.744358)
Supplement: Supplementary file 1 [file DataSheet_1.docx]

**Supplementary material**

**HIVEP1 is a negative regulator of NF-κB that inhibits systemic inflammation in sepsis**

Hisatake Matsumoto^1^, Brendon P. Scicluna^1,2^, Kin Ki Jim^3,4^, Fahimeh Falahi^1^, Wanhai Qin^1^, Berke Gürkan^1^, Erik Malmstrӧm^1^, Mariska T. Meijer^1^, Joe Butler^1^, Hina N. Khan^1^, Tsuyoshi Takagi^5^, Shunsuke Ishii^6^, Marcus J. Schultz^7,8,9^, Diederik van de Beek^4^, Alex F. de Vos^1^, Cornelis van ’t Veer^1^, Tom van der Poll^1,10*^

1. Center for Experimental and Molecular Medicine, Amsterdam University Medical Centers, location Academic Medical Center, University of Amsterdam, Amsterdam, The Netherlands
2. Department of Clinical Epidemiology, Biostatistics and Bioinformatics, Amsterdam University Medical Centers, location Academic Medical Center, University of Amsterdam, Amsterdam, The Netherlands
3. Department of Medical Microbiology and Infection Prevention, Amsterdam University Medical Centers, location Academic Medical Center, University of Amsterdam, Amsterdam, The Netherlands.
4. Department of Neurology, Amsterdam University Medical Centers, location Academic Medical Center, University of Amsterdam, Amsterdam, The Netherlands
5. Department of Disease Model, Institute for Developmental Research, Aichi

Developmental Disability Center, Kasugai, Aichi, Japan

1. RIKEN Cluster for Pioneering Research, Tsukuba, Ibaraki, Japan
2. Department of Intensive Care Medicine, and Laboratory of Experimental Intensive Care and Anesthesiology (L·E·I·C·A), Amsterdam University Medical Centers, location Academic Medical Center, University of Amsterdam, Amsterdam, The Netherlands
3. Mahidol-Oxford Tropical Medicine Research Unit (MORU), Mahidol University, Bangkok, Thailand
4. Nuffield Department of medicine, University of Oxford, Oxford, UK
5. Division of Infectious Diseases, Amsterdam University Medical Centers, location Academic Medical Center, University of Amsterdam, Amsterdam, The Netherlands

*** Correspondence:**Tom van der Poll. Academic Medical Center, Meibergdreef 9, Room G2-130, 1105AZ Amsterdam, the Netherlands. Phone: +31-20-5665910.

Mail: [t.vanderpoll@amsterdamumc.nl](mailto:t.vanderpoll@amsterdamumc.nl)

**Figure S1.**

**Targeted disruption of *hivep1* in mice**

**(a)** Schematic representation of the *hivep1* allele; the targeting vector and predicted disrupted allele are shown. To disrupt the *hivep1* gene, part of exon 4 was replaced by a neo cassette.

**(b)** Homologous recombination with targeting vector on the *hivep1* allele was examined by Southern blot analysis using with genomic DNA from mouse ES cells (left panel). The 5’ side recombination on the *hivep1* allele was confirmed by Apa I digestion with 5’ probe, which discriminates wild type and mutant allele, 5.0 kb and 4.7 kb, respectively (right panel). The 3’ side recombination was confirmed by BamH I digestion with 3’ probe, which discriminates wild type and mutant allele, 12.0 kb and 8.5 kb, respectively.

**(c)** Result of genotyping PCR performed on genomic DNA from mouse tails is shown.

**(d)** RT-PCR analysis using primers in exon 3 & 4 of HIVEP1 (and HPRT) of bone marrow derived macrophages from hivep1-/- mice and wild type littermates. Bone marrow derived macrophages from *hivep1^-/-^* mice and wild type littermates were stimulated with LPS (100 ng/mL) for 2 and 8 hours. *Hivep1^-/-^* macrophages showed decreased expression of HIVEP1 mRNA upon LPS stimulation. Data are means with standard error of the mean and were pooled from three independent data set (n=4 replicates samples for each condition within each experiment). ***P < 0.001.

**
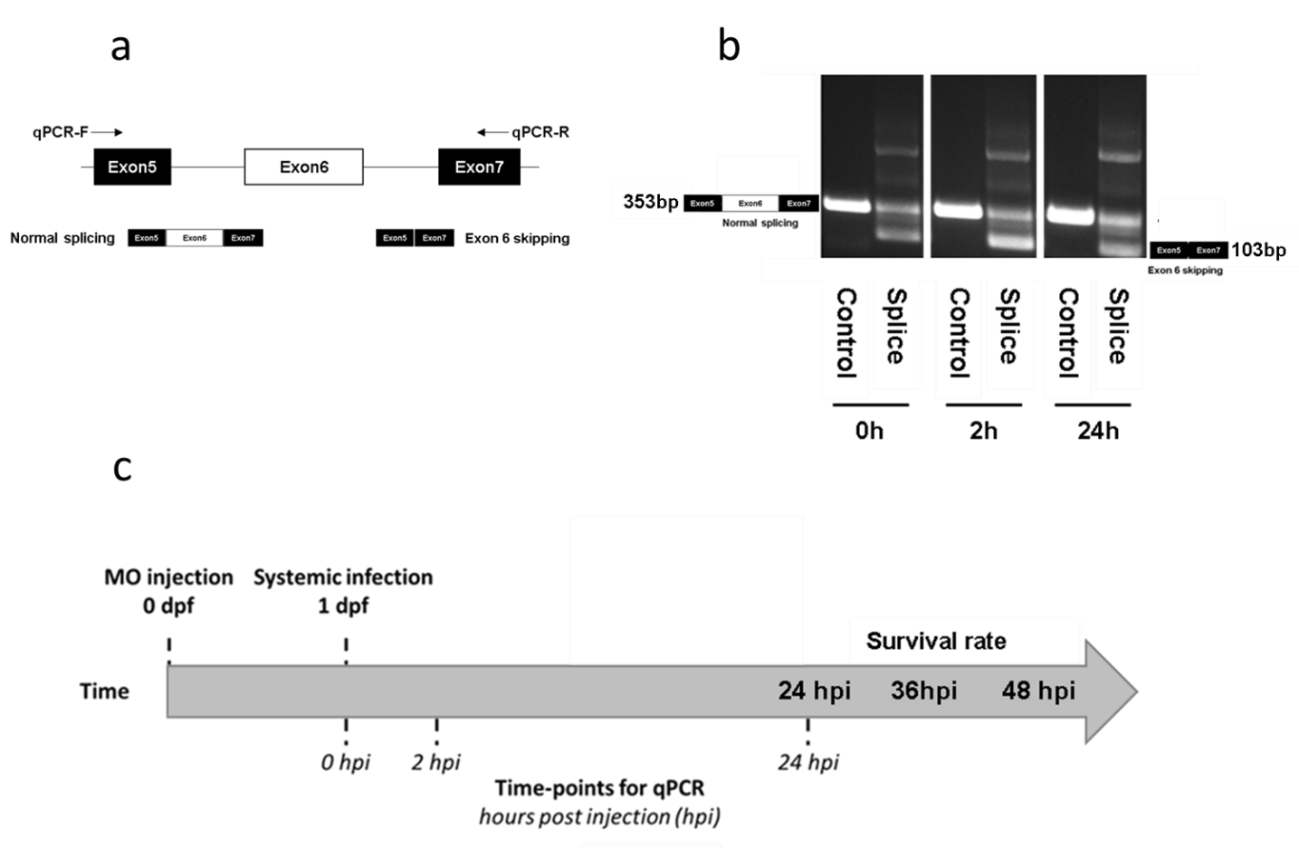
**

**Figure S2.**

**HIVEP1 gene knockdown in *Danio rerio* using splice-modifying antisense morpholino oligonucleotides**

**(a)** HIVEP1 splice-modifying antisense morpholino oligonucleotide (H1MO) was designed to prevent correct splicing of *Danio rerio* HIVEP1 by targeting exon 6 from the HIVEP1 mRNA. The forward and reverse primers (arrows) were used to test the efficacy of H1MO. Standard control morpholino oligo (SCMO) was predicted to lead to a normal splicing product (353 bps).

**(b)** Verification of the efficacy of H1MO. Comparison of control lane and splice lane indicate that H1MO reduce the level of correctly spliced transcript and leads to alternatively spliced products at 0, 2, and 24 hours post fertilization. The RT-qPCR products of HIVEP1 mRNA were loaded into a 2% agarose gel. The comparison of control lane and splice lane indicates that H1MO induced exon 6 skipping products (103 bp) and products of weak cryptic splice sites. **(c)** Experimental protocol of zebrafish infection experiments.

**Figure S3.**

**Generation of HIVEP1 deficient THP1-MD2-CD14 cells by CRISPR/Cas9**

(a) Schematic representation of the *HIVEP1* gene. Guide sequences for CRISPR/Cas9-mediated mutagenesis in exon 3 and 4 and location of primers for (q) PCR are indicated.

(b) Result of genomic DNA PCR analysis using primers specific for sequences flanking exon 2, 3, 4 and 5 of *HIVEP1* and for *HPRT* for two control clones and one clone targeted at exon 3 and exon 4. The HIVEP1 deficient THP1-MD2-CD14 cells show bands for HPRT, and HIVEP1 exon 2 and 5, but not for exon 3 and exon 4. (c) Result of RT-PCR analysis using primers in exon 3 & 4, and exon 8 & 9 of *HIVEP1* (and *HPRT*) of one control clone and one clone targeted at exon 3 and exon 4 stimulated for 8 hours with medium or LPS. HIVEP1 expression was detected in THP1-MD2-CD14 cells under basal conditions and was induced by LPS (P<0.001). HIVEP1 expression using primers in exon 3 & 4 showed that HIVEP1 was not induced by LPS in HIVEP1 targeted THP1-MD2-CD14 cells. The sequences of exon3 and exon4 targeted by guide RNA (gRNA) are shown in Red. PCR-F1/R1and PCR-F2/R2 primers are for PCR amplifications of the HIVEP1 exon 3 and exon 4 respectively. qPCR-F1/R1 and qPCR-F2/R2 are primers for qPCR of HIVEP1 exon3_4 and exon8_9 mRNA respectively.

**Figure. S4: Cotransfection of HIVEP-1 does not affect MyD88 or TRAF6 expression.**

HEK293T cells were transfected with MyD88 (a) and TRAF6 (b) to induce NF-κB as in Fig2e-f with control empty vector or HIVEP-1 vector. MyD88 and TRAF6 expression was determined by western blot.

|  | | | |
| --- | --- | --- | --- |
| **Species** | **Gene** | **Oligo Name** | **Sequence (5' > 3')** |
| **Human** | **Primers used for PCR amplification** | | |
|  | HPRT |  | TGAGTTTGGAAACATCTGGAGT |
|  |  |  | AAAGGGAACTGCTGACAAAGA |
|  | HIVEP1 exon2 | PCR-F2 | ATGGAGCTGCCTTTTGGTGG |
|  |  | PCR-R2 | TGGGATGAATTTGTTTAGTTCGAGG |
|  | HIVEP1 exon3 | PCR-F3 | AGCTGTAGTTCAGTATCTAAAGTGT |
|  |  | PCR-R3 | TAAGGAACACAGTTGGCCGT |
|  | HIVEP1 exon4 | PCR-F4 | CGCAAAAAGATCGTAGCTGAGAA |
|  |  | PCR-R4 | TCAACAGCTTCCGGGGTGTT |
|  | HIVEP1 exon5 | PCR-F5 | GCATTAGGTAATCAAAAGTCCACAG |
|  |  | PCR-R5 | TCTTCTTGGTTCACTTTTGATTAAGGA |
|  | **Primers used for CHIP-qPCR** | | |
|  | TNF promoter | CCCTCCAGTTCTAGTTCTATC | GGGGAAAGAATCATTCAACCA |
|  | TNFAIP3 promoter | CAGCCCGACCCAGAGAGT | GGGGGTGTGATCTCTCTTGG |
|  | **Primers used for RT-qPCR** | | |
|  | **Gene** | **Forward** | **Reverse** |
|  | HPRT | GGATTTGAAATTCCAGACAAGTTT | GCGATGTCAATAGGACTCCAG |
|  | HIVEP1 exon3_4 | ACAAAATTGAAGAAGCACAAAAAG | AGCAACCATCTGAACTGCGTAGA |
|  | HIVEP1 exon8_9 | TGTGTGGATTTAGGCGTCTC | TGTGGCTGACAGGACTGATT |
|  | TNF-α | CAGGGACCTCTCTCTAATCAGC | GCTGGTTATCTCTCAGCTCCAC |
|  | IL1-β | CGCAGGACAGGTACAGATTCTT | AAAAAGCTTGGTGATGTCTGGT |
|  | IL-6 | AACATGTGTGAAAGCAGCAAAG | CTCTCAAATCTGTTCTGGAGGT |
|  | IL-8 | AACCTTTCCACCCCAAATTTAT | AAAACTTCTCCACAACCCTCTG |
|  | A-20 | TCCAGAACACCATTCCGTG | TGAGGTGCTTTGTGTGGTTC |
|  |  |  |  |
| **Mouse** | **Gene** | **Oligo Name** | **Sequence (5' > 3')** |
|  | **Primers used for PCR amplification** | | |
|  | HIVEP1 intron4-exon4 | PCR-FW1 | AACAGAAGTCTTAGAGGCTGGCG |
|  | HIVEP1 exon4 | PCR-R1 | GGATTCCTCAGGGGGGATTTTGG |
|  | HIVEP1 exon4 | PCR-R2 | AAGAAGGGTGAGAACAGAGTACC |
|  | **Primers used for RT-qPCR** | | |
|  | **Gene** | **Forward** | **Reverse** |
|  | HPRT | AGTCAAGGGCATATCCAACA | CAAACTTTGCTTTCCGGGT |
|  | HIVEP1 exon3_4 | ATCGAAGAAGCACAAAAAGAGCT | GCTCGGGAGGACACTGAAC |
|  | TNF-α | CGAGTGACAAGCCTGTAGCC | CCTTGAAGAGAACCTGGGAGT |
|  | IL1-β | GGGGAACTCTGCAGACTCAA | GGGCCTCAAAGGAAAGAATC |
|  | IL-6 | CTTCCTACCCCAATTTCCAATGCT | TCTTGGTCCTTAGCCACTCCTT |
|  | CXCL1 | CCACTGCACCCAAACCGAAG | TCCGTTACTTGGGGACACCT |
|  |  |  |  |
| **Zebrafish** | **Primers used for RT-qPCR** | | |
|  | **Gene** | **Forward** | **Reverse** |
|  | mobk13 | CACCCGTTTCGTGATGAAGTACAA | GTTAAGCAGGATTTACAATGGAG |
|  | HIVEP1 Exon5_7 | ACAGAGACACTTCCAAATCGGA | CTGAGTCTTCTGCGTCCTGG |
|  | TNF-α | GCTGGATCTTCAAAGTCGGGTGTA | TGTGAGTCTCAGCACACTTCCATC |
|  | IL1-β | TGGACTTCGCAGCACAAAATG | GTTCACTTCACGCTCTTGGATG |
|  | CXCL8a | GTCGCTGCATTGAAACAGAA | CTTAACCCATGGAGCAGAGG |
|  | CXCL8b | CTACCGAGACGTGGGTGATT | GCTCGGTGAATGGTCATTTT |

**Table S1.**

**Primers used for PCR amplification**

| **Upregulated canonical pathways** | **adjusted p-value (-log)** |
| --- | --- |
| Mitochondrial Dysfunction | 2.65 |
| Reelin Signaling in Neurons | 2.65 |
| Ethanol Degradation IV | 2.65 |
| Oxidative Phosphorylation | 2.65 |
|  |  |
| **Downregulated canonical pathways** | **adjusted p-value (-log)** |
| Interferon Signaling | 4.64 |
| Role of Macrophages, Fibroblasts and Endothelial Cells in Rheumatoid Arthritis | 3.5 |
| Systemic Lupus Erythematosus In B Cell Signaling Pathway | 3.5 |
| Crosstalk between Dendritic Cells and Natural Killer Cells | 3.07 |
| Dendritic Cell Maturation | 3.06 |
| Type I Diabetes Mellitus Signaling | 3.03 |
| Phagosome Formation | 3.03 |
| Hepatic Fibrosis Signaling Pathway | 2.95 |
| Th1 and Th2 Activation Pathway | 2.78 |
| Role of Pattern Recognition Receptors in Recognition of Bacteria and Viruses | 2.77 |
| Communication between Innate and Adaptive Immune Cells | 2.58 |
| Hepatic Fibrosis / Hepatic Stellate Cell Activation | 2.58 |
| IL-10 Signaling | 2.46 |
| Altered T Cell and B Cell Signaling in Rheumatoid Arthritis | 2.46 |
| T Cell Exhaustion Signaling Pathway | 2.46 |
| Caveolar-mediated Endocytosis Signaling | 2.21 |
| IL-9 Signaling | 2.21 |
| Death Receptor Signaling | 2.09 |
| Pathogenesis of Multiple Sclerosis | 2.04 |

**Table S2**. **Ingenuity pathway analysis of differentially expressed genes in HIVEP1 deficient cells relative to controls at baseline.** Adjusted p-value, Adjusted p-value, Fisher’s exact test and Benjamini-Hochberg correction.

| **Upregulated canonical pathways** | **adjusted p-value (-log)** |
| --- | --- |
| Phagosome Formation | 10.9 |
| Role of Pattern Recognition Receptors in Recognition of Bacteria and Viruses | 10.3 |
| Dendritic Cell Maturation | 10 |
| Systemic Lupus Erythematosus In B Cell Signaling Pathway | 9.74 |
| Neuroinflammation Signaling Pathway | 9.03 |
| Altered T Cell and B Cell Signaling in Rheumatoid Arthritis | 8.41 |
| TREM1 Signaling | 8.05 |
| Role of Macrophages, Fibroblasts and Endothelial Cells in Rheumatoid Arthritis | 7.73 |
| Th1 and Th2 Activation Pathway | 7.48 |
| Hepatic Fibrosis / Hepatic Stellate Cell Activation | 7.42 |
| Communication between Innate and Adaptive Immune Cells | 7.4 |
| T Helper Cell Differentiation | 6.87 |
| Cardiac Hypertrophy Signaling (Enhanced) | 6.84 |
| Hepatic Fibrosis Signaling Pathway | 6.81 |
| Granulocyte Adhesion and Diapedesis | 6.76 |
| Hepatic Cholestasis | 6.28 |
| IL-10 Signaling | 6.27 |
| Type I Diabetes Mellitus Signaling | 6.09 |
| T Cell Exhaustion Signaling Pathway | 6.04 |
| Phospholipase C Signaling | 5.92 |
| Th1 Pathway | 5.84 |
| Toll-like Receptor Signaling | 5.65 |
| Role of NFAT in Regulation of the Immune Response | 5.64 |
| TNFR2 Signaling | 5.47 |
| Role of Tissue Factor in Cancer | 5.43 |
| IL-6 Signaling | 5.27 |
| Interferon Signaling | 5.07 |
| HMGB1 Signaling | 5.04 |
| Production of Nitric Oxide and Reactive Oxygen Species in Macrophages | 4.73 |
| Th2 Pathway | 4.73 |
| Inflammasome pathway | 4.68 |
| Tec Kinase Signaling | 4.43 |
| NF-κB Signaling | 4.4 |
| Cholecystokinin/Gastrin-mediated Signaling | 4.39 |
| Antioxidant Action of Vitamin C | 4.33 |
| IL-8 Signaling | 4.2 |
| Role of IL-17A in Arthritis | 4.08 |
| Agranulocyte Adhesion and Diapedesis | 4.03 |
| Graft-versus-Host Disease Signaling | 4.02 |
| PPARα/RXRα Activation | 3.94 |
| Xenobiotic Metabolism Signaling | 3.92 |
| Sperm Motility | 3.82 |
| PKCθ Signaling in T Lymphocytes | 3.79 |
| IL-17A Signaling in Gastric Cells | 3.75 |
| Leukocyte Extravasation Signaling | 3.63 |
| PPAR Signaling | 3.62 |
| Pathogenesis of Multiple Sclerosis | 3.62 |
| Acute Phase Response Signaling | 3.6 |
| Thrombin Signaling | 3.59 |
| Renin-Angiotensin Signaling | 3.56 |
| OX40 Signaling Pathway | 3.56 |
| NF-κB Activation by Viruses | 3.51 |
| PI3K Signaling in B Lymphocytes | 3.47 |
| UVA-Induced MAPK Signaling | 3.46 |
| iNOS Signaling | 3.46 |
| Endothelin-1 Signaling | 3.42 |
| CD40 Signaling | 3.36 |
| Role of PKR in Interferon Induction and Antiviral Response | 3.34 |
| CD28 Signaling in T Helper Cells | 3.29 |
| Colorectal Cancer Metastasis Signaling | 3.28 |
| CXCR4 Signaling | 3.2 |
| Role of Hypercytokinemia/hyperchemokinemia in the Pathogenesis of Influenza | 3.16 |
| Systemic Lupus Erythematosus In T Cell Signaling Pathway | 3.12 |
| Crosstalk between Dendritic Cells and Natural Killer Cells | 3.08 |
| G-Protein Coupled Receptor Signaling | 3.06 |
| Death Receptor Signaling | 3.03 |
| Erythropoietin Signaling | 3.01 |
| iCOS-iCOSL Signaling in T Helper Cells | 2.98 |
| IL-1 Signaling | 2.98 |
| Sphingosine-1-phosphate Signaling | 2.97 |
| IL-12 Signaling and Production in Macrophages | 2.95 |
| Atherosclerosis Signaling | 2.91 |
| Natural Killer Cell Signaling | 2.81 |
| Glioma Invasiveness Signaling | 2.81 |
| IL-15 Production | 2.77 |
| PD-1, PD-L1 cancer immunotherapy pathway | 2.74 |
| Endocannabinoid Neuronal Synapse Pathway | 2.73 |
| Calcium-induced T Lymphocyte Apoptosis | 2.69 |
| GNRH Signaling | 2.66 |
| IL-4 Signaling | 2.66 |
| Regulation of Cytokine Production in Intestinal Epithelial Cells  by IL-17A and IL-17F | 2.65 |
| Integrin Signaling | 2.64 |
| Activation of IRF by Cytosolic Pattern Recognition Receptors | 2.64 |
| B Cell Activating Factor Signaling | 2.64 |
| Cytokine Production in Macrophages and T Helper Cells by IL-17A and IL-17F | 2.64 |
| B Cell Receptor Signaling | 2.63 |
| Allograft Rejection Signaling | 2.6 |
| fMLP Signaling in Neutrophils | 2.58 |
| Adrenomedullin signaling pathway | 2.51 |
| Semaphorin Signaling in Neurons | 2.44 |
| GPCR-Mediated Nutrient Sensing in Enteroendocrine Cells | 2.38 |
| Gαq Signaling | 2.36 |
| p70S6K Signaling | 2.36 |
| Caveolar-mediated Endocytosis Signaling | 2.35 |
| 4-1BB Signaling in T Lymphocytes | 2.32 |
| LXR/RXR Activation | 2.31 |
| April Mediated Signaling | 2.26 |
| Lymphotoxin β Receptor Signaling | 2.24 |
| Axonal Guidance Signaling | 2.23 |
| LPS/IL-1 Mediated Inhibition of RXR Function | 2.23 |
| Germ Cell-Sertoli Cell Junction Signaling | 2.22 |
| Role of NFAT in Cardiac Hypertrophy | 2.2 |
| Protein Kinase A Signaling | 2.1 |
| STAT3 Pathway | 2.09 |
| Role of Osteoblasts, Osteoclasts and Chondrocytes in Rheumatoid Arthritis | 2.08 |
| IL-17A Signaling in Fibroblasts | 2.06 |
| Autoimmune Thyroid Disease Signaling | 2.04 |
| B Cell Development | 2.03 |
| GPCR-Mediated Integration of Enteroendocrine Signaling Exemplified  by an L Cell | 2 |
| CTLA4 Signaling in Cytotoxic T Lymphocytes | 2 |
| Regulation of IL-2 Expression in Activated and Anergic T Lymphocytes | 2 |
| PI3K/AKT Signaling | 2 |
|  |  |
| **Downregulated canonical pathways** | **adjusted p-value (-log)** |
| Oxidative Phosphorylation | 21.6 |
| Mitochondrial Dysfunction | 18.9 |
| Sirtuin Signaling Pathway | 13.3 |
| Cell Cycle Control of Chromosomal Replication | 10.1 |
| NER Pathway | 8.45 |
| Superpathway of Cholesterol Biosynthesis | 7.09 |
| Mismatch Repair in Eukaryotes | 5.98 |
| Cholesterol Biosynthesis I | 5.97 |
| Cholesterol Biosynthesis II (via 24,25-dihydrolanosterol) | 5.97 |
| Cholesterol Biosynthesis III (via Desmosterol) | 5.97 |
| Mitotic Roles of Polo-Like Kinase | 5.87 |
| Cell Cycle: G2/M DNA Damage Checkpoint Regulation | 5.35 |
| Hereditary Breast Cancer Signaling | 5.09 |
| EIF2 Signaling | 3.4 |
| Estrogen-mediated S-phase Entry | 2.86 |
| Protein Ubiquitination Pathway | 2.79 |
| DNA Methylation and Transcriptional Repression Signaling | 2.68 |
| Pyrimidine Deoxyribonucleotides De Novo Biosynthesis I | 2.68 |
| RAN Signaling | 2.65 |
| Role of BRCA1 in DNA Damage Response | 2.56 |
| Role of CHK Proteins in Cell Cycle Checkpoint Control | 2.54 |

**Table S3**. **Ingenuity pathway analysis of differentially expressed genes in HIVEP1 deficient cells relative to controls after 2 hours LPS-stimulation.** Adjusted p-value, Adjusted p-value, Fisher’s exact test and Benjamini-Hochberg correction.

| **Upregulated canonical pathways** | **adjusted p-value (-log)** |
| --- | --- |
| Th1 and Th2 Activation Pathway | 12.8 |
| Neuroinflammation Signaling Pathway | 12.8 |
| Dendritic Cell Maturation | 11.8 |
| TREM1 Signaling | 11.6 |
| Role of Pattern Recognition Receptors in Recognition of Bacteria and Viruses | 11 |
| Type I Diabetes Mellitus Signaling | 11 |
| Hepatic Fibrosis Signaling Pathway | 11 |
| Th2 Pathway | 10.4 |
| IL-10 Signaling | 9.59 |
| Systemic Lupus Erythematosus In B Cell Signaling Pathway | 9.44 |
| T Cell Exhaustion Signaling Pathway | 9.13 |
| T Helper Cell Differentiation | 8.9 |
| Toll-like Receptor Signaling | 8.72 |
| Hepatic Fibrosis / Hepatic Stellate Cell Activation | 8.59 |
| Inflammasome pathway | 8.54 |
| Communication between Innate and Adaptive Immune Cells | 8.5 |
| Altered T Cell and B Cell Signaling in Rheumatoid Arthritis | 8.37 |
| Crosstalk between Dendritic Cells and Natural Killer Cells | 8.27 |
| Th1 Pathway | 8.16 |
| Natural Killer Cell Signaling | 7.98 |
| Role of PKR in Interferon Induction and Antiviral Response | 7.93 |
| Interferon Signaling | 7.93 |
| Role of Macrophages, Fibroblasts and Endothelial Cells in Rheumatoid Arthritis | 7.88 |
| Death Receptor Signaling | 7.57 |
| Phagosome Formation | 7.36 |
| HMGB1 Signaling | 7.26 |
| iNOS Signaling | 7.16 |
| Acute Phase Response Signaling | 7.12 |
| OX40 Signaling Pathway | 6.85 |
| PD-1, PD-L1 cancer immunotherapy pathway | 6.78 |
| Production of Nitric Oxide and Reactive Oxygen Species in Macrophages | 6.63 |
| Graft-versus-Host Disease Signaling | 6.39 |
| IL-6 Signaling | 6.39 |
| NF-κB Signaling | 6.36 |
| Tec Kinase Signaling | 6.11 |
| TNFR2 Signaling | 5.97 |
| Antigen Presentation Pathway | 5.87 |
| TWEAK Signaling | 5.81 |
| Granulocyte Adhesion and Diapedesis | 5.78 |
| Systemic Lupus Erythematosus In T Cell Signaling Pathway | 5.57 |
| Osteoarthritis Pathway | 5.27 |
| IL-8 Signaling | 5.1 |
| TNFR1 Signaling | 5.05 |
| Cholecystokinin/Gastrin-mediated Signaling | 5.05 |
| IL-12 Signaling and Production in Macrophages | 5.04 |
| Hepatic Cholestasis | 4.9 |
| Role of IL-17A in Arthritis | 4.83 |
| Activation of IRF by Cytosolic Pattern Recognition Receptors | 4.76 |
| Cardiac Hypertrophy Signaling (Enhanced) | 4.63 |
| Molecular Mechanisms of Cancer | 4.46 |
| CD40 Signaling | 4.44 |
| Allograft Rejection Signaling | 4.41 |
| B Cell Development | 4.31 |
| STAT3 Pathway | 4.27 |
| Unfolded protein response | 4.22 |
| IL-17A Signaling in Fibroblasts | 4.12 |
| Apoptosis Signaling | 4.12 |
| Colorectal Cancer Metastasis Signaling | 4.12 |
| iCOS-iCOSL Signaling in T Helper Cells | 4.01 |
| Role of Hypercytokinemia/hyperchemokinemia in the Pathogenesis of Influenza | 4 |
| Autoimmune Thyroid Disease Signaling | 3.87 |
| 4-1BB Signaling in T Lymphocytes | 3.86 |
| Induction of Apoptosis by HIV1 | 3.72 |
| p38 MAPK Signaling | 3.66 |
| Necroptosis Signaling Pathway | 3.65 |
| JAK/Stat Signaling | 3.58 |
| PI3K Signaling in B Lymphocytes | 3.54 |
| Phagosome Maturation | 3.54 |
| Pathogenesis of Multiple Sclerosis | 3.49 |
| IL-17A Signaling in Gastric Cells | 3.49 |
| Role of JAK family kinases in IL-6-type Cytokine Signaling | 3.49 |
| PPAR Signaling | 3.49 |
| CD28 Signaling in T Helper Cells | 3.47 |
| Protein Kinase A Signaling | 3.47 |
| Erythropoietin Signaling | 3.44 |
| B Cell Receptor Signaling | 3.44 |
| Role of Tissue Factor in Cancer | 3.42 |
| PI3K/AKT Signaling | 3.42 |
| IL-15 Production | 3.3 |
| IL-1 Signaling | 3.3 |
| Role of NFAT in Regulation of the Immune Response | 3.3 |
| Agranulocyte Adhesion and Diapedesis | 3.3 |
| Phospholipase C Signaling | 3.25 |
| PPARα/RXRα Activation | 3.21 |
| IL-17 Signaling | 3.17 |
| LXR/RXR Activation | 3.15 |
| Differential Regulation of Cytokine Production in Intestinal Epithelial Cells  by IL-17A and IL-17F | 3.11 |
| IL-23 Signaling Pathway | 3.11 |
| LPS-stimulated MAPK Signaling | 3.04 |
| IL-4 Signaling | 3.04 |
| Integrin Signaling | 3.04 |
| Xenobiotic Metabolism Signaling | 3.02 |
| Gαq Signaling | 3.01 |
| Role of JAK1, JAK2 and TYK2 in Interferon Signaling | 2.97 |
| CD27 Signaling in Lymphocytes | 2.93 |
| FAT10 Cancer Signaling Pathway | 2.93 |
| IL-17A Signaling in Airway Cells | 2.88 |
| B Cell Activating Factor Signaling | 2.86 |
| Role of IL-17F in Allergic Inflammatory Airway Diseases | 2.86 |
| Fcγ Receptor-mediated Phagocytosis in Macrophages and Monocytes | 2.79 |
| Calcium-induced T Lymphocyte Apoptosis | 2.79 |
| Role of RIG1-like Receptors in Antiviral Innate Immunity | 2.77 |
| Cdc42 Signaling | 2.77 |
| Pancreatic Adenocarcinoma Signaling | 2.74 |
| Atherosclerosis Signaling | 2.71 |
| Ceramide Signaling | 2.7 |
| Acute Myeloid Leukemia Signaling | 2.7 |
| Regulation of IL-2 Expression in Activated and Anergic T Lymphocytes | 2.7 |
| Th17 Activation Pathway | 2.7 |
| Oncostatin M Signaling | 2.7 |
| Renin-Angiotensin Signaling | 2.67 |
| Role of JAK2 in Hormone-like Cytokine Signaling | 2.66 |
| NRF2-mediated Oxidative Stress Response | 2.63 |
| Antioxidant Action of Vitamin C | 2.6 |
| IL-9 Signaling | 2.55 |
| April Mediated Signaling | 2.53 |
| PKCθ Signaling in T Lymphocytes | 2.53 |
| Sperm Motility | 2.52 |
| Germ Cell-Sertoli Cell Junction Signaling | 2.51 |
| Sphingosine-1-phosphate Signaling | 2.47 |
| Cytokine Production in Macrophages and T Helper Cells by IL-17A and IL-17F | 2.47 |
| MIF-mediated Glucocorticoid Regulation | 2.46 |
| RANK Signaling in Osteoclasts | 2.44 |
| Leukocyte Extravasation Signaling | 2.39 |
| ILK Signaling | 2.39 |
| Nur77 Signaling in T Lymphocytes | 2.39 |
| IL-7 Signaling Pathway | 2.39 |
| Chondroitin and Dermatan Biosynthesis | 2.39 |
| GP6 Signaling Pathway | 2.39 |
| Role of JAK1 and JAK3 in γc Cytokine Signaling | 2.35 |
| Thrombin Signaling | 2.34 |
| Prolactin Signaling | 2.34 |
| CXCR4 Signaling | 2.31 |
| TGF-β Signaling | 2.31 |
| G-Protein Coupled Receptor Signaling | 2.31 |
| MIF Regulation of Innate Immunity | 2.3 |
| IL-15 Signaling | 2.3 |
| PEDF Signaling | 2.3 |
| Dermatan Sulfate Biosynthesis | 2.28 |
| Glucocorticoid Receptor Signaling | 2.22 |
| VDR/RXR Activation | 2.19 |
| Actin Nucleation by ARP-WASP Complex | 2.19 |
| GM-CSF Signaling | 2.19 |
| Myc Mediated Apoptosis Signaling | 2.18 |
| Semaphorin Signaling in Neurons | 2.15 |
| Gα12/13 Signaling | 2.13 |
| γ-glutamyl Cycle | 2.13 |
| fMLP Signaling in Neutrophils | 2.13 |
| Endoplasmic Reticulum Stress Pathway | 2.1 |
| IL-3 Signaling | 2.09 |
| Glioma Invasiveness Signaling | 2.08 |
| Type II Diabetes Mellitus Signaling | 2.06 |
| Chondroitin Sulfate Biosynthesis | 2.05 |
| Senescence Pathway | 2.05 |
| Caveolar-mediated Endocytosis Signaling | 2.03 |
|  |  |
| **Downregulated canonical pathways** | **adjusted p-value (-log)** |
| Mitochondrial Dysfunction | 7.44 |
| Oxidative Phosphorylation | 6.66 |
| Superpathway of Cholesterol Biosynthesis | 5.82 |
| Cell Cycle Control of Chromosomal Replication | 4.91 |
| Isoleucine Degradation I | 3.71 |
| Cholesterol Biosynthesis I | 3.28 |
| Cholesterol Biosynthesis II (via 24,25-dihydrolanosterol) | 3.28 |
| Cholesterol Biosynthesis III (via Desmosterol) | 3.28 |
| Pyrimidine Deoxyribonucleotides De Novo Biosynthesis I | 2.45 |
| Valine Degradation I | 2.24 |

**Table S4**. **Ingenuity pathway analysis of differentially expressed genes in HIVEP1 deficient cells relative to controls after 8 hours LPS-stimulation.** Adjusted p-value, Adjusted p-value, Fisher’s exact test and Benjamini-Hochberg correction.
